# Risk factors associated with postoperative necrosis after digital replantation: a systematic review and meta-analysis

**DOI:** 10.3389/fsurg.2026.1795247

**Published:** 2026-04-13

**Authors:** Feiyong Yu, Lingjing Lu, Yitong Zhou, Ruiying Wang, Hong Zheng

**Affiliations:** 1 Department of Trauma and Extremity Orthopedics, The First Affiliated Hospital of Guilin Medical University, Guilin, Guangxi Zhuang Autonomous Region, China; 2 Department of Emergency Medicine, The First Affiliated Hospital of Guilin Medical University, Guilin, Guangxi Zhuang Autonomous Region, China; 3 Guangxi Key Laboratory of Diabetic Systems Medicine, Guilin Medical University, Guilin, Guangxi Zhuang Autonomous Region, China; 4 Thyroid Surgery, The First Affiliated Hospital of Guilin Medical University, Guilin, Guangxi Zhuang Autonomous Region, China; 5 Department of Dermatology, The First Affiliated Hospital of Guilin Medical University, Guilin, Guangxi Zhuang Autonomous Region, China; 6 Department of Geriatrics, The First Affiliated Hospital of Guilin Medical University, Guilin, Guangxi Zhuang Autonomous Region, China

**Keywords:** finger replantation, meta-analysis, postoperative necrosis, related factors, systematic review

## Abstract

**Background:**

Systematically evaluate and quantitatively analyze risk factors associated with necrosis following replantation surgery for amputated fingers, providing evidence-based support for perioperative risk assessment and clinical decision-making.

**Methods:**

Systematically searched PubMed, Embase, Web of Science, Cochrane Library, and CNKI, Wan fang, VIP for all observational studies from indexing to January 1, 2026. Cohort studies reporting overall postoperative necrosis after finger replantation, as defined in the original studies, and its associated factors were included. The Newcastle–Ottawa Scale (NOS) was used to assess the methodological quality of included studies. Pooled effect sizes were extracted, and pooled odds ratios (ORs) with 95% confidence intervals (95% CI) were calculated using a random-effects model.

**Results:**

A total of 12 studies involving 3,645 patients were included. Meta-analysis results suggest smoking [OR = 3.48, 95% CI (1.96, 6.17)], preoperative ischemic time ≥8 h [OR = 2.25, 95% CI (1.43, 3.54)], crush injury [OR = 2.77, 95% CI (1.41, 5.43)], thrombosis [OR = 4.98, 95% CI (1.49, 16.67)], vein graft [OR = 1.56, 95% CI (1.19, 2.04)] may be associated with necrosis after digital replantation

**Conclusion:**

This study suggests that smoking, preoperative ischemia duration ≥8 h, crush injury, thrombosis, and venous grafting may be associated with an increased risk of overall postoperative necrosis following finger replantation. These findings should be interpreted with caution because outcome definitions were not fully consistent across studies.

**Systematic Review Registration:**

https://www.crd.york.ac.uk/PROSPERO/view/CRD420261290184.

## Background

Digital replantation stands as one of the most representative emergency reconstructive procedures in hand surgery and microsurgery, aiming to restore the patient's hand to its maximum anatomical integrity and functional capacity (1). With continuous advancements in microsurgical techniques, anesthesia management, and perioperative care, the overall success rate of digital replantation has significantly improved compared to previous eras (2). However, postoperative complications remain relatively common, with circulatory compromise and subsequent necrosis of the replanted digit being the most severe (3). These complications not only directly impact the success of the replantation but may also increase the risk of reoperation, infection, or even amputation, imposing substantial physical, psychological, and financial burdens on patients (4).

Postoperative necrosis is generally considered the primary manifestation of failed replantation. Its pathogenesis is complex and often closely associated with arterial or venous blood flow obstruction (5). In clinical practice, even with advanced vascular anastomosis techniques and standardized surgical procedures, some patients may still experience vascular crises postoperatively, progressing to irreversible tissue necrosis (6). This phenomenon suggests that necrosis after finger replantation is not caused by a single factor but rather results from the combined effects of multiple injury-related factors, patient-specific factors, and surgical-related factors (7).

Previous studies (8, 9) indicate that the type of injury plays a significant role in replantation prognosis. Compared to sharp lacerations, crush injuries and avulsion injuries often involve more severe soft tissue contusions, vascular endothelial damage, and microcirculatory impairment, significantly increasing the risk of replantation failure (10). Furthermore, prolonged ischemia is widely recognized as closely associated with tissue hypoxia and reperfusion injury. This is particularly true during excessive cold or warm ischemia periods, which can readily induce vasospasm, thrombosis, and postoperative necrosis (11). Factors such as multiple finger injuries or proximal injury planes may also compromise graft survival by increasing surgical complexity and vascular reconstruction difficulty.

Beyond the injury itself, the patient's underlying health status can significantly influence replantation outcomes. Smoking is associated with vascular endothelial dysfunction and hypercoagulability, potentially elevating risks of postoperative vascular thrombosis and necrosis. while underlying conditions such as diabetes and hypertension may reduce replantation success rates by impairing microcirculation and wound healing capacity (12, 13). Currently, studies on factors associated with necrosis after finger replantation are predominantly single-center, small-sample observational studies, yielding inconsistent results and lacking systematic evaluation of the combined effects of various factors. Some studies (14, 15) focus solely on single factors, failing to adequately consider the potential for multifactorial interactions. This limitation restricts the generalizability of findings to clinical practice. Therefore, a systematic review and meta-analysis is necessary to provide clinicians with more reliable evidence-based guidance for preoperative risk assessment, intraoperative decision-making, and postoperative management. Such an analysis would also inform the design of future high-quality prospective studies.

## Methods

This study was designed and reported in strict adherence to the Preferred Reporting Items for Systematic Reviews and Meta-Analyses (PRISMA) guidelines (16), with the registration number: CRD420261290184.

### Literature search strategy

Systematic searches were conducted in PubMed, Embase, Web of Science, Cochrane Library, and relevant Chinese databases (CNKI, Wan fang, VIP), covering the period from each database's inception to January 1, 2026. A consistent search framework combining subject headings and free-text terms was applied across all databases. The search strategy was adapted as necessary according to the specific characteristics and indexing systems of each database to ensure sensitivity and comprehensiveness. Key English search terms included “digital replantation,” “finger replantation,” “necrosis,” and “risk factors,” with adjustments made according to each database's characteristics. Reference lists of included studies were also traced to minimize potential omissions. The detailed search strategy is presented in Supplementary Table S1.

### Inclusion and exclusion criteria

#### Inclusion criteria

Study subjects are patients undergoing replantation surgery for amputated fingers.
Study types are observational studies, including cohort studies and case-control studies.

Reports postoperative necrosis outcomes following finger replantation surgery and analyzes at least one potential associated factor. Because definitions were not fully uniform across studies, the outcome was extracted as overall postoperative necrosis as reported by the original authors, regardless of whether complete necrosis and partial necrosis were separately distinguished.

Provides effect size data suitable for meta-analysis, such as odds ratio (OR) with 95% confidence interval (CI), or raw data enabling calculation of these metrics.

#### Exclusion criteria

Review articles, case reports, conference abstracts, animal studies, or basic research.
Studies with unclear subjects or those not reporting data specifically for replanted finger patients.
Studies with poorly defined outcome measures or those from which valid data cannot be extracted.

Duplicate publications—only the study with the largest sample size or most complete information will be retained.

Studies involving revascularization procedures (without complete amputation) or incomplete amputations were excluded where this information was explicitly reported, as these conditions may differ from complete digital replantation in terms of prognosis and underlying mechanisms.

### Data extraction

Two researchers independently screened the literature. Initial screening was based on titles and abstracts, followed by full-text review to determine final inclusion. Disagreements were resolved through discussion with a third researcher. A pre-designed Excel data extraction form was used to collect relevant information, including: first author, publication year, country of study, study design, sample size, patient characteristics (gender, mean age), and regression models. In addition, we extracted information on the definition of postoperative necrosis used in each study, including whether necrosis was reported as an overall outcome or explicitly classified as complete or partial necrosis.

### Quality assessment

This study employed the Newcastle–Ottawa Scale (NOS) (17) to evaluate the methodological quality of included observational studies. The NOS was selected because it is a widely used and validated tool for assessing the quality of cohort and case–control studies and is considered appropriate for systematic reviews of observational research. This scale systematically assesses research quality across three dimensions: Selection, Comparability, and Outcome, with a maximum total score of 9 points. Regarding subject selection, the evaluation focuses on the representativeness of cases and controls (or exposed vs. unexposed cohorts), the method of determining exposure factors or outcome measures, and whether clear and reasonable criteria were used for subject inclusion. For comparability, the assessment primarily evaluates whether potential confounding factors (age, injury type, or comorbidities) were controlled or adjusted for. For outcome assessment, the focus is on the method used to determine postoperative necrosis, the adequacy of follow-up duration, and the handling of loss to follow-up. Two researchers independently conducted the quality assessments. Discrepancies in scoring were resolved through discussion and negotiation, with a third researcher involved for adjudication when necessary. Based on the NOS scores, study quality was categorized as high quality (7–9 points), moderate quality (4–6 points), and low quality (≤3 points).

### Data analysis

Given the variability in outcome definitions among included studies, pooled analyses were conducted using the overall postoperative necrosis outcome as reported in the original articles. Statistical analysis was performed using Stata 15.0 software. Odds ratios (OR) and their 95% confidence intervals (95% CI) were used as effect size measures to assess the association between potential factors and the occurrence of necrosis following finger replantation surgery. Considering potential clinical and methodological differences among included studies in terms of design, population characteristics, injury mechanisms, and outcome definitions, a random-effects model was uniformly applied for effect size pooling. Heterogeneity among studies was assessed using Cochran's *Q* test and the I^2^ statistic, where I^2^ < 25% indicated low heterogeneity, 25%–50% represented moderate heterogeneity, and >50% signified substantial heterogeneity. When high heterogeneity was observed in the pooled analysis of a specific factor, sensitivity analysis and meta-regression was further conducted to explore the sources of heterogeneity. This involved sequentially excluding individual studies to observe changes in the pooled effect size and I^2^ statistic, thereby assessing the stability of results and the influence of individual studies on the overall findings. Publication bias is preliminarily assessed using funnel plots, followed by quantitative evaluation via Egger's regression test. A *P*-value <0.05 suggests potential publication bias. All statistical tests are two-tailed, with *P* < 0.05 considered statistically significant.

## Results

### Literature search results

**Figure 1:**
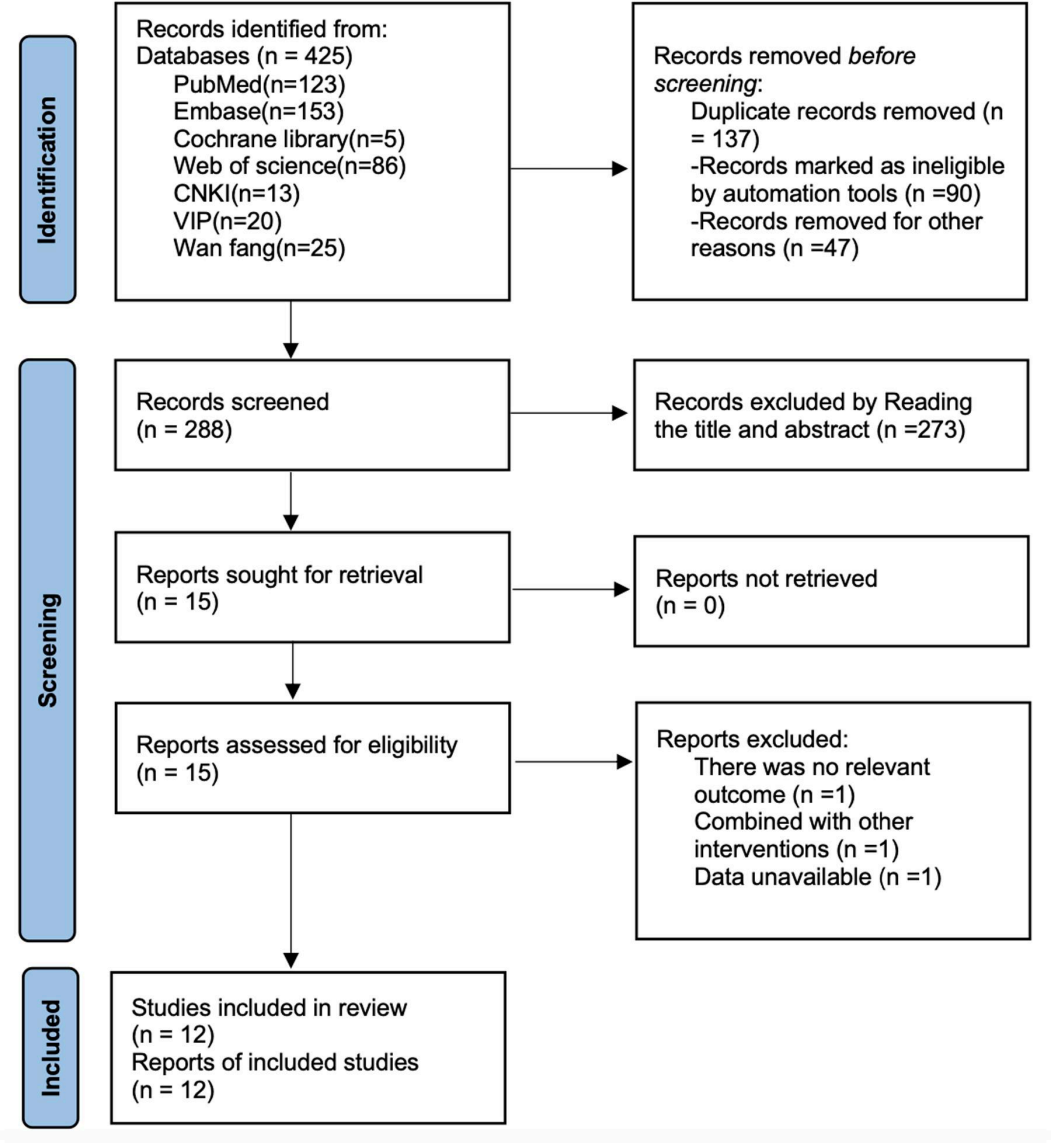
Literature search flow chart.

As shown in Figure 1, a total of 425 articles were retrieved through searches of PubMed (*n* = 123), Embase (*n* = 153), Cochrane Library (*n* = 5), Web of Science (*n* = 86), CNKI (*n* = 13), VIP (*n* = 20), Wan fang (*n* = 25), a total of 425 articles were retrieved. After removing 137 duplicate publications, 273 articles were excluded based on title and abstract screening, and 3 full-text articles were discarded. Ultimately, 12 articles (18–29) were included for analysis.

### Basic characteristics included study

This study included a total of 12 articles involving 3,645 patients, with a necrosis incidence rate of 15.1% (552/3645). The included studies were published between 2016 and 2025 and were all cohort studies. All subjects were patients who underwent replantation surgery for amputated fingers. The studies primarily originated from Asia, with subjects ranging in age from 35 to 60 years. Detailed characteristics of the included studies are presented in Table 1.

**Table 1:** Basic characteristics of the included studies.

Cohort study									
Study	Representativeness of the exposed group	Selection of non-exposed groups	Determination of exposure factors	Identification of outcome indicators not yet to be observed at study entry	Comparability of exposed and unexposed groups considered in design and statistical analysis	design and statistical analysis	Adequacy of the study's evaluation of the outcome	Adequacy of follow-up in exposed and unexposed groups	Total scores
Cai 2025	*	*	*	*	**	*	*	*	9
Guo 2024	*	*	*	/	**	*	*	*	8
Jin 2019	*	*	*	/	**	*	*	*	8
Nishijima 2016	*	*	*	*	**	*	*	*	9
Pang 2025	*	*	*	*	**	*	*	*	9
Peng 2023	*	*	*	/	*	*	*	*	7
Wang 2023	*	*	*	/	**	*	*	*	8
GL Feng 2021	*	*	*	/	*	*	*	*	7
XR Zhou 2023	*	*	*	/	**	*	*	*	8
PJ Zhang 2021	*	*	*	/	*	*	*	*	7
YD Li 2025	*	*	*	*	**	*	*	*	9
CB Wei 2023	*	*	*	/	*	*	*	*	7

* One scores.

/, Not applicable.

### Risk of bias results

The NOS was used to assess the quality of the included studies. Results showed that the NOS scores ranged from 7 to 9 points, with 12 studies classified as high-quality (7–9 points). Overall, the included studies demonstrated high quality in subject selection and outcome assessment, though some studies exhibited certain shortcomings in controlling for confounding factors. The quality assessment results are presented in Table 2.

**Table 2:** NOS results.

Study	Year	Country	Study design	Sample size	Number of necrosis	Gender(M/F)	Postoperative necrosis	Mean age(years)	Regression model
Cai	2025	China	Cohort study	67	13	45/22	Overall	40	Logistic regression
Guo	2024	China	Cohort study	332	54	244/88	Overall	43.1	Logistic regression
Jin	2019	China	Cohort study	134	12	115/19	Overall	35	Logistic regression
Nishijima	2016	Japan	Cohort study	144	16	130/14	Complete/partial necrosis	44	Logistic regression
Pang	2025	China	Cohort study	201	18	148/53	Overall	43.6	Logistic regression
Peng	2023	China	Cohort study	378	69	310/68	Complete/partial necrosis	39.9	Logistic regression
Wang	2023	China	Cohort study	946	201	669/277	Overall	42.5	Logistic regression
GL Feng	2021	China	Cohort study	120	8	89/31	Overall	60	Logistic regression
XR Zhou	2023	China	Cohort study	184	42	161/23	Overall	36.24	Logistic regression
PJ Zhang	2021	China	Cohort study	240	40	156/84	Overall	43.1	Logistic regression
YD Li	2025	China	Cohort study	658	34	527/131	Overall	39.4	Logistic regression
CB Wei	2023	China	Cohort study	241	45	146/95	Overall	35.5	Logistic regression

## Meta-analysis results

### Smoking

**Figure 2:**
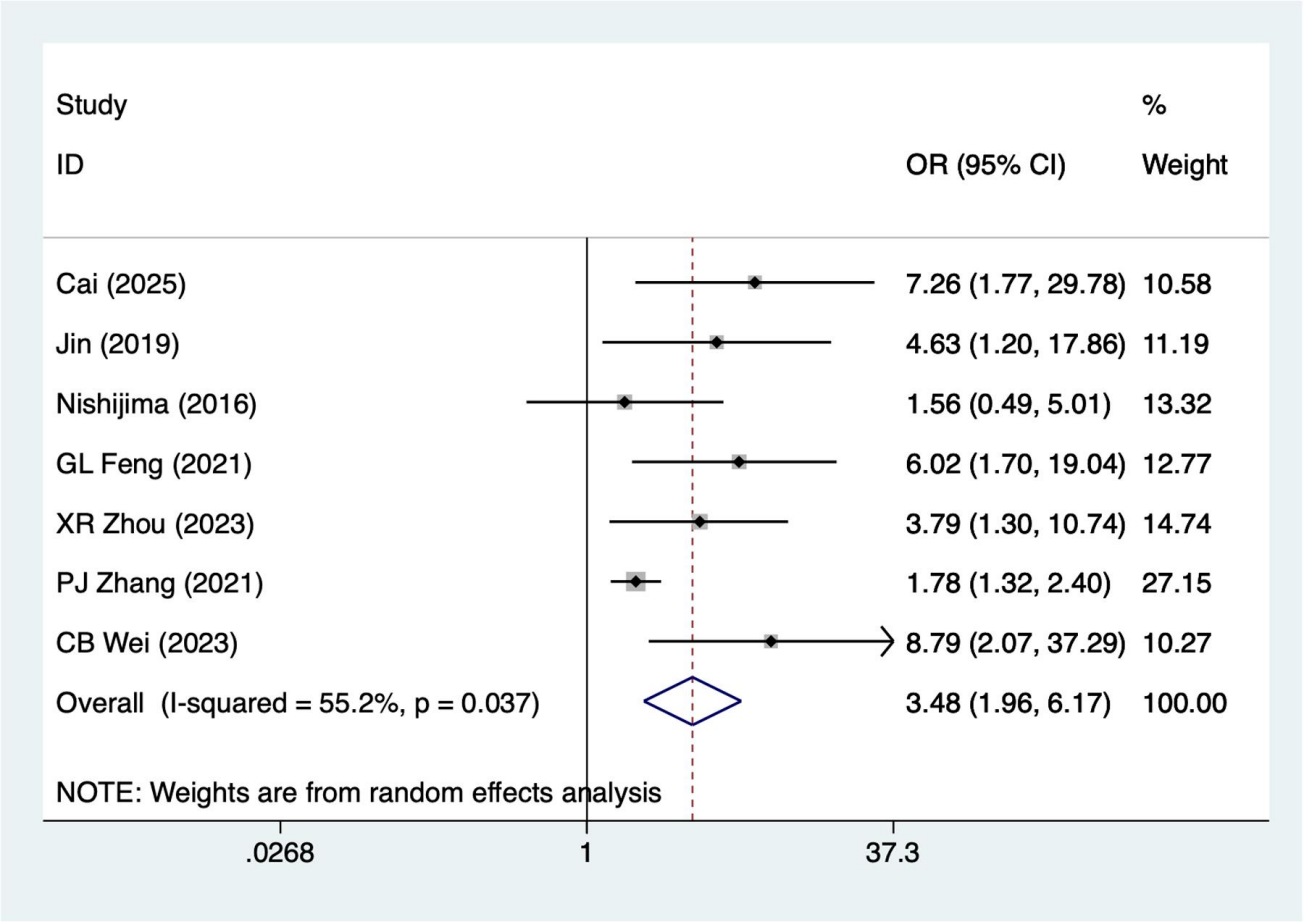
Forest plot of the meta-analysis on smoking.

7 studies mentioned smoking. A random-effects model was used for heterogeneity testing (I^2^ = 55.2%, *P* = 0.037). Analysis results (Figure 2) suggest smoking may be associated with necrosis after digital replantation [OR = 3.48, 95% CI (1.96, 6.17)]. Sensitivity analysis (Supplementary Figure S1) suggests heterogeneity likely originates from PJ Zhang 2021.

### Preoperative ischemic time ≥8h

**Figure 3:**
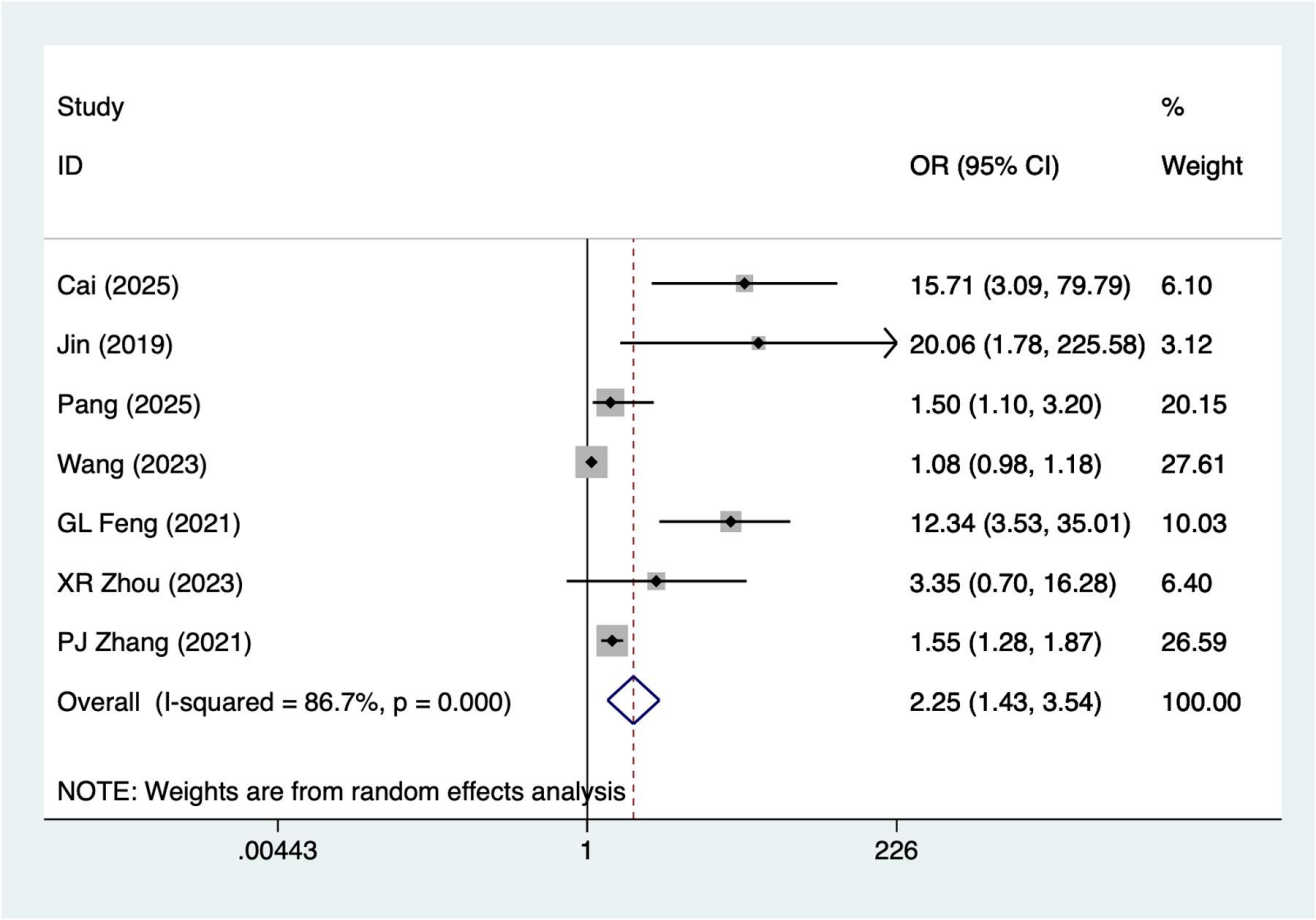
Forest plot of the meta-analysis on preoperative ischemic time ≥8h.

7 studies mentioned preoperative ischemic time ≥8 h. A random-effects model was used for heterogeneity testing (I^2^ = 86.7%, *P* = 0.001). Analysis results (Figure 3) suggest preoperative ischemic time ≥8 h may be associated with necrosis after digital replantation [OR = 2.25, 95% CI (1.43, 3.54)]. Sensitivity analysis (Supplementary Figure S2) suggests stable results unaffected by any single study.

### Crush injury

**Figure 4:**
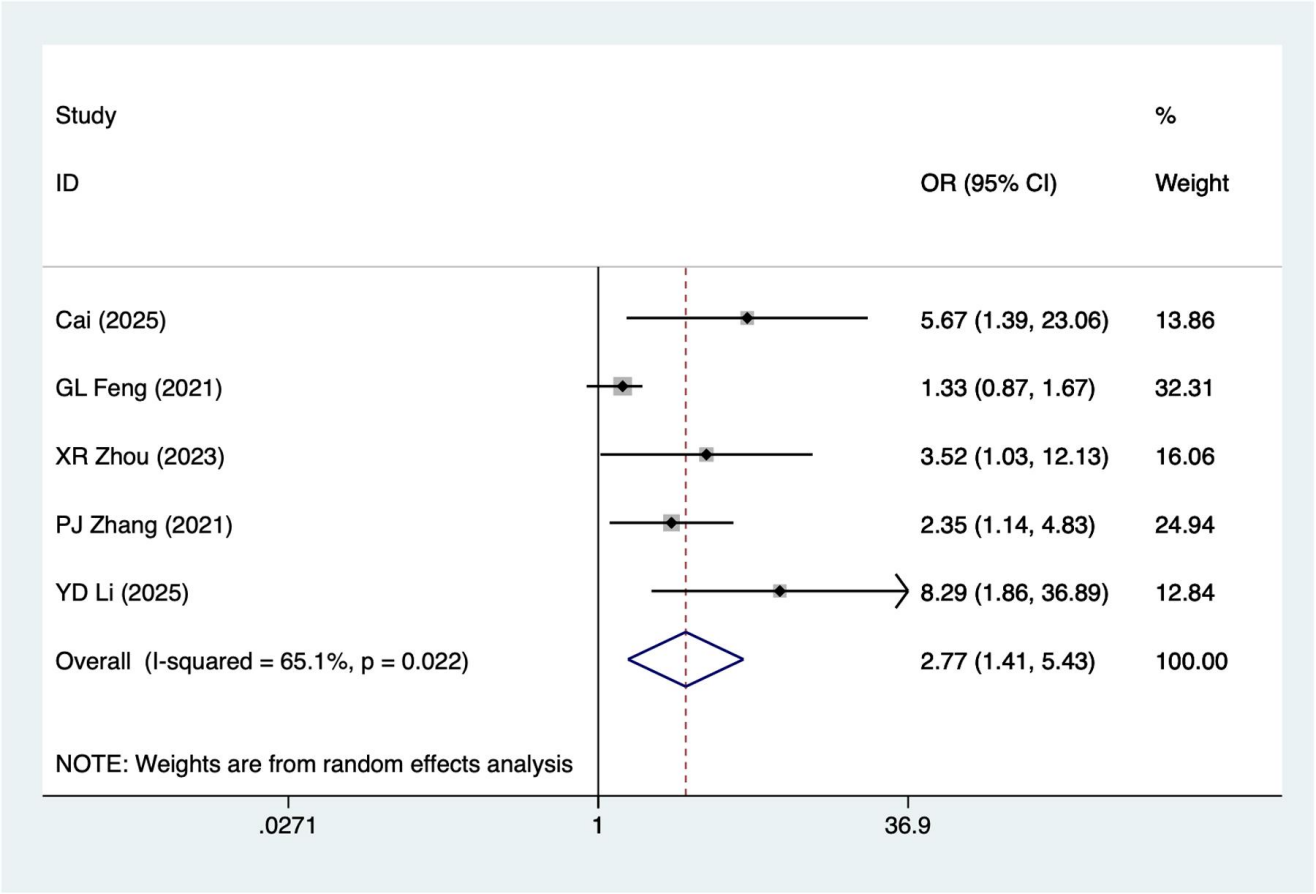
Forest plot of the meta-analysis on crush injury.

5 studies mentioned crush injury. A random-effects model was used for heterogeneity testing (I^2^ = 65.1%, *P* = 0.022). Analysis results (Figure 4) suggest crush injury may be associated with necrosis after digital replantation [OR = 2.77, 95% CI (1.41, 5.43)]. Sensitivity analysis (Supplementary Figure S3) suggests heterogeneity likely originates from GL Feng 2021.

### Thrombosis

**Figure 5:**
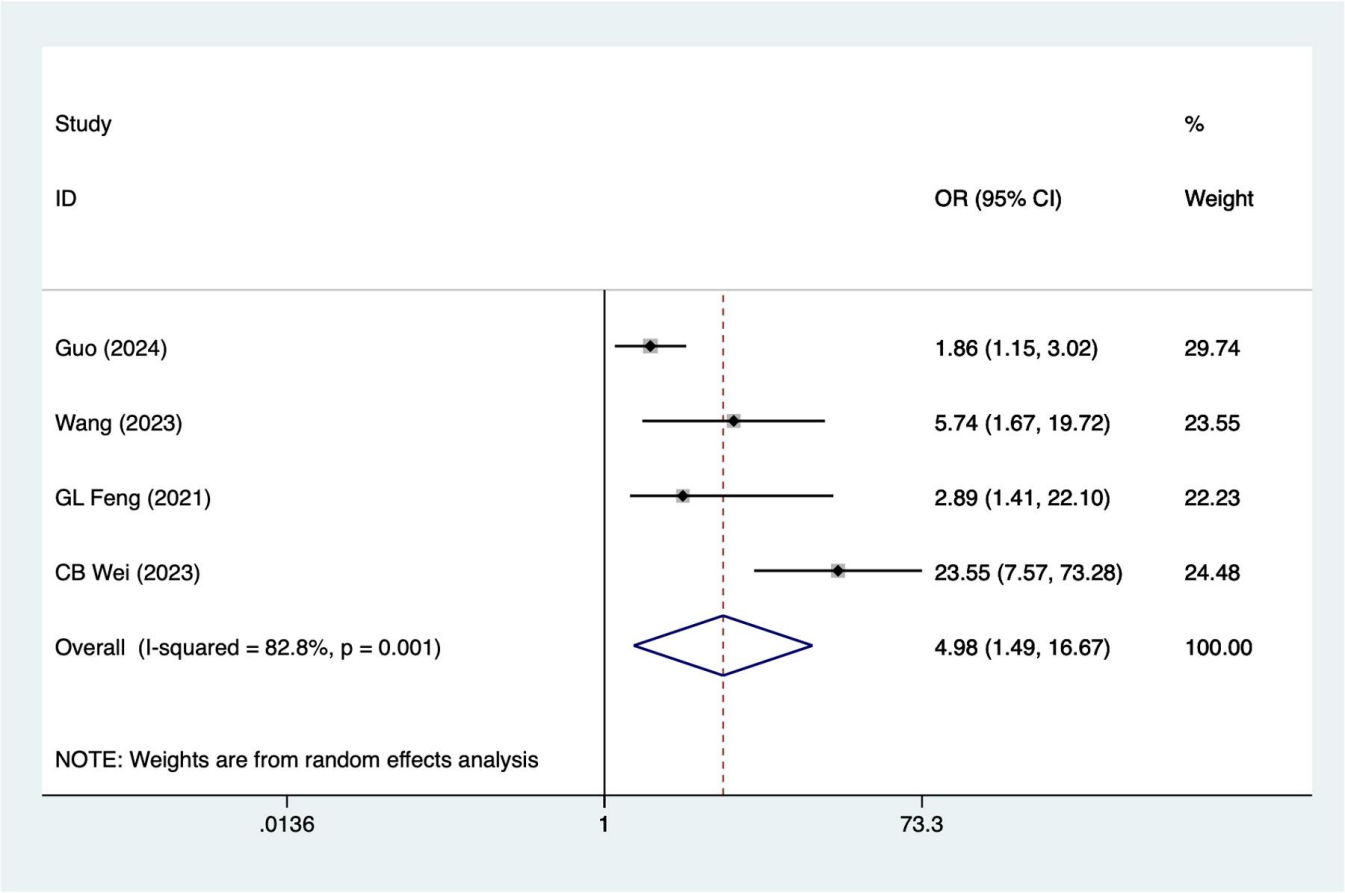
Forest plot of the meta-analysis on thrombosis.

4 studies mentioned thrombosis. A random-effects model was used for heterogeneity testing (I^2^ = 82.8%, *P* = 0.001). Analysis results (Figure 5) suggest thrombosis may be associated with necrosis after digital replantation [OR = 4.98, 95% CI (1.49, 16.67)]. Sensitivity analysis (Supplementary Figure S4) suggests heterogeneity likely originates from Guo 2024.

### Vein graft

**Figure 6:**
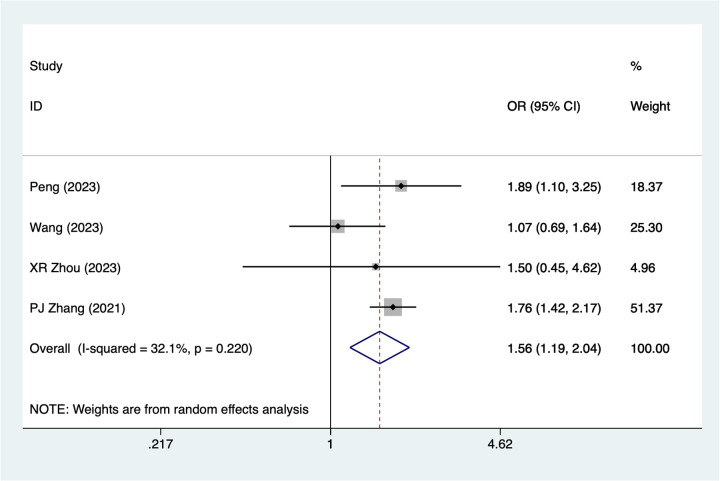
Forest plot of the meta-analysis on vein graft.

Four studies reported on vein graft use. A random-effects model was applied (I^2^ = 32.1%, *P* = 0.220). The pooled analysis (Figure 6) suggests that vein graft use may be associated with an increased risk of postoperative necrosis after digital replantation [OR = 1.56, 95% CI (1.19, 2.04)].

### Meta-regression

Meta-regression (Table 3) analyses showed that none of the examined covariates, including mean age, publication year, country, study design, and postoperative necrosis rate, may be associated with effect size across all risk factors (all *P* > 0.05).

**Table 3:** Meta-regression analysis results.

Covariate	Smoking *β* (P)	Ischemic time ≥8 h *β* (P)	Crush injury *β* (P)	Thrombosis *β* (P)	Vein graft *β* (P)
Mean age	0.65 (0.120)	0.11 (0.260)	0.45 (0.230)	0.26 (0.770)	0.51 (0.270)
Publication year	−0.26 (0.650)	0.87 (0.170)	0.33 (0.750)	0.56 (0.450)	0.37 (0.240)
Country	0.26 (0.170)	0.91 (0.540)	0.23 (0.260)	0.34 (0.750)	0.43 (0.810)
Study design	0.86 (0.410)	−0.27 (0.130)	0.11 (0.370)	0.56 (0.340)	0.36 (0.280)
Postoperative necrosis	0.21 (0.310)	0.37 (0.190)	0.34 (0.470)	0.27 (0.440)	0.19 (0.340)

β coefficients were derived from univariable meta-regression models. No statistically significant associations were identified (all *P* > 0.05).

### Publication bias

This study assessed publication bias using the Egger test and funnel plots (Supplementary Figures S5-S9). Results indicated funnel plot asymmetry for smoking (*P* = 0.001), preoperative ischemic time ≥8 h (*P* = 0.01), and crush injury (*P* = 0.002), suggesting a high likelihood of publication bias. In contrast, funnel plots for thrombosis (*P* = 0.13) and vein graft (*P* = 0.65) showed symmetry, indicating a low likelihood of publication bias. Results from the trim-and-fill analysis for smoking, preoperative ischemic time ≥8 h, and crush injury (Supplementary Figures S10-12) indicate that the findings remain stable even when accounting for publication bias.

## Discussion

In this meta-analysis, we identified several factors that may be associated with an increased risk of postoperative necrosis following finger replantation, including smoking, prolonged preoperative ischemia (≥8 h), crush injury, thrombosis, and the use of vein grafts. These findings highlight the multifactorial nature of replantation outcomes and underscore the importance of perioperative risk assessment and optimization. However, given the observational nature of the included studies and potential residual confounding, these associations should be interpreted with caution.

A previous meta-analysis by Ma et al. (2016) (30) evaluated factors influencing the survival rate of digital replantation, and their findings regarding the adverse impact of crush or avulsion injuries are broadly consistent with our results. However, their analysis mainly relied on unadjusted (single factor) estimates, whereas the present study preferentially used adjusted results from multivariable analyses, which may better account for potential confounding. In addition, while survival rate reflects an overall outcome, our study focuses on postoperative necrosis as a more specific clinical endpoint related to vascular compromise.

This study demonstrates that smokers face a significantly increased risk of postoperative necrosis, suggesting smoking may be a key factor affecting the survival of replanted digits (31). The primary mechanism by which smoking elevates necrosis risk likely involves its effects on vascular function and microcirculation. Smoking impairs vascular endothelial function, induces vasospasm and reduces blood flow, while simultaneously increasing blood viscosity and platelet aggregation tendency, promoting thrombus formation and exacerbating blood flow obstruction in the replanted digit (32, 33). Additionally, long-term smoking induces atherosclerosis and microvascular lesions, diminishing tissue tolerance to ischemia and reperfusion injury (34). In clinical practice, assessing smoking history serves as a critical reference for perioperative risk stratification. High-risk patients should receive enhanced preoperative smoking cessation counseling, optimized intraoperative vascular anastomosis techniques, and rigorous postoperative anticoagulation management.

Ischemia time is a significant predictor of successful replantation of amputated fingers. This study demonstrates that patients with preoperative ischemia time ≥8 h exhibit a markedly elevated risk of necrosis. Prolonged ischemia leads to local tissue hypoxia, accumulation of metabolic byproducts and lactic acid, disrupts cell membrane stability, and induces oxidative stress, thereby exacerbating microcirculatory damage (35). Additionally, reperfusion injury may occur during revascularization, including endothelial dysfunction and local inflammatory responses, further increasing the risk of thrombosis and tissue necrosis (36, 37). Therefore, for patients with prolonged ischemia, clinicians should strive to minimize ischemia duration whenever possible. When necessary, hypothermic preservation should be employed to delay tissue metabolism. Postoperatively, vascular patency should be closely monitored, with prompt intervention upon early detection of blood flow obstruction.

Crush injuries represent another significant factor in this study, suggesting that crush or avulsion injuries are more likely to cause postoperative necrosis than sharp lacerations. Crush injuries typically involve extensive soft tissue damage, vascular endothelial injury, and microvascular disruption, leading to hemodynamic abnormalities and increased risk of thrombosis (38, 39). During replantation surgery, such injuries often necessitate more complex vascular repair and debridement procedures, increasing surgical difficulty. They may also lead to postoperative vasospasm or impaired venous return, thereby affecting digit survival rates (40). Clinically, patients with crush injuries should undergo enhanced preoperative assessment, thorough debridement, and measures to ensure adequate venous return.

Thrombosis is one of the direct mechanisms leading to necrosis following replantation surgery. This study demonstrates that patients with postoperative thrombosis exhibit a significantly elevated risk of necrosis, indicating a close association between vascular occlusion and replantation failure (41). Thrombosis can obstruct arteries or veins, cause persistent local tissue ischemia and subsequently induce phalangeal necrosis. The causes of thrombosis may be related to intraoperative vascular injury, endothelial dysfunction, hypercoagulable states, and ischemia-reperfusion injury (42). Therefore, meticulous intraoperative techniques, thorough thrombus clearance, and postoperative anticoagulation management are key measures to reduce necrosis risk.

Our findings suggest a slightly higher risk of postoperative necrosis in patients undergoing venous grafting. This association may be related to technical complexity and the potential for thrombosis or flow obstruction at anastomotic sites, which could compromise vascular patency (43). However, this finding should be interpreted with caution. The use of vein grafts often reflects greater injury severity or more extensive vascular damage and may therefore act as a surrogate marker of case complexity rather than an independent risk factor. Consequently, the observed association may be influenced by residual confounding.

In addition, several clinically relevant factors may influence replantation outcomes but were not sufficiently reported for quantitative analysis in the present study. These include multiple finger replantation, perioperative anticoagulation strategies (heparin use), and patient comorbidities such as diabetes and vascular disease. These factors may affect microcirculation, thrombosis risk, and overall tissue viability. The lack of consistent reporting across studies limited further exploration of their effects, highlighting the need for more comprehensive and standardized data collection in future research.

## Clinical significance

The findings of this study suggest that high-risk patients should be prioritized for perioperative management. For smokers, preoperative smoking cessation counseling should be intensified. For patients with prolonged anticipated ischemia time, severe injuries, or those requiring vein graft, surgical plans should be optimized and postoperative blood flow closely monitored. For patients experiencing thrombosis or circulatory compromise, timely intervention is essential. By comprehensively addressing these multifactorial considerations, the success rate of finger replantation can be enhanced, the risk of necrosis reduced, and consequently, patients’ functional recovery and quality of life improved. These findings are broadly consistent with current clinical understanding of replantation outcomes, further supporting their potential relevance in perioperative risk stratification.

## Strengths and limitations

The primary strengths of this study are as follows: First, it systematically integrated multiple observational studies and quantitatively assessed various factors associated with necrosis following finger replantation through meta-analysis, yielding results with greater statistical reliability and reference value. Second, the included studies spanned different countries and regions with substantial sample sizes, comprehensively reflecting the prevalent characteristics of necrosis after finger replantation in clinical practice. Third, the study employed strict inclusion criteria and methodological quality assessment (NOS scale), supplemented by analyses of heterogeneity, sensitivity, and publication bias, enhancing the robustness and credibility of the findings. Furthermore, these results provide evidence-based guidance for perioperative risk assessment, intraoperative decision-making, and postoperative monitoring, facilitating individualized management for high-risk patients.

However, this study also has certain limitations: First, all included studies were observational designs, potentially introducing confounding factors and selection bias, preventing the establishment of clear causal relationships; Second, the definition of postoperative necrosis was not fully consistent across studies. Most studies reported necrosis as an overall postoperative outcome, whereas only a minority explicitly differentiated complete necrosis from partial necrosis. This inconsistency may have introduced clinical heterogeneity, limited the precision of pooled estimates, and reduced the ability to draw more nuanced conclusions regarding the severity of necrotic outcomes. In addition, differences in ischemia time grouping and criteria for assessing vascular complications may also have contributed to heterogeneity; Third, the limited number of studies addressing certain key factors (thrombosis, venous grafting) restricted the ability to conduct subgroup analyses and further explore interactions. Finally, the exclusion of unpublished or gray literature introduces potential publication bias. Future prospective, multicenter, large-scale studies are needed to validate the independent effects and interactions of these associated factors.

## Conclusion

This study suggests that smoking, preoperative ischemia duration ≥8 h, crush injury, thrombosis, and venous grafting may be associated with an increased risk of overall postoperative necrosis following finger replantation. However, because definitions of necrosis were not fully standardized across studies, these findings should be interpreted cautiously and confirmed in future prospective studies using uniform outcome criteria.

## Data Availability

The original contributions presented in the study are included in the article/Supplementary Material, further inquiries can be directed to the corresponding authors.
