# Supplementary material for: Risk factors associated with postoperative necrosis after digital replantation: a systematic review and meta-analysis
Source: Front Surg. 2026 Apr 13;13:1795247. doi: 10.3389/fsurg.2026.1795247 (PMC13111292; doi:10.3389/fsurg.2026.1795247)
Supplement: Supplementary file 1 [file Datasheet1.docx]

Table S1 search strategy

**PUBMED:**

((("Fingers"[Mesh]) OR ((((Fingers[Title/Abstract]) OR (Finger[Title/Abstract])) OR (Thumb[Title/Abstract])) OR (Digital[Title/Abstract]))) AND (("Replantation"[Mesh]) OR ((((((((Replantation[Title/Abstract]) OR (Replantations[Title/Abstract])) OR (Reimplantation[Title/Abstract])) OR (Reimplantations[Title/Abstract])) OR (Replantation, Surgical[Title/Abstract])) OR (Replantations, Surgical[Title/Abstract])) OR (Surgical Replantations[Title/Abstract])) OR (Surgical Replantation[Title/Abstract])))) AND (("Necrosis"[Mesh]) OR (Necrosis[Title/Abstract]))

**Embase**

| No. | Query | Results |
| --- | --- | --- |
| #20 | #6 AND #16 AND #19 | 153 |
| #19 | #17 OR #18 | 1310620 |
| #18 | 'necrosis':ab,ti | 467932 |
| #17 | 'necrosis'/exp | 987978 |
| #16 | #7 OR #8 OR #9 OR #10 OR #11 OR #12 OR #13 OR #14 OR #15 | 22943 |
| #15 | 'surgical replantation':ab,ti | 35 |
| #14 | 'surgical replantations':ab,ti | 0 |
| #13 | 'replantations, surgical':ab,ti | 0 |
| #12 | 'replantation, surgical':ab,ti | 10 |
| #11 | 'reimplantations':ab,ti | 609 |
| #10 | 'reimplantation':ab,ti | 13681 |
| #9 | 'replantations':ab,ti | 649 |
| #8 | 'replantation':ab,ti | 5347 |
| #7 | 'reimplantation'/exp | 13427 |
| #6 | #1 OR #2 OR #3 OR #4 OR #5 | 475184 |
| #5 | 'digital':ab,ti | 305109 |
| #4 | 'thumb':ab,ti | 24615 |
| #3 | 'finger':ab,ti | 117815 |
| #2 | 'fingers':ab,ti | 41715 |
| #1 | 'finger'/exp | 58451 |

**Cochrane**

ID Search Hits

#1 MeSH descriptor: [Fingers] explode all trees 1162

#2 (Fingers):ti,ab,kw OR (Finger):ti,ab,kw OR (Thumb):ti,ab,kw OR (Digital):ti,ab,kw 38133

#3 #1or#2 38133

#4 MeSH descriptor: [Replantation] explode all trees 68

#5 (Replantation):ti,ab,kw OR (Replantations):ti,ab,kw OR (Reimplantation):ti,ab,kw OR (Reimplantations):ti,ab,kw OR (Replantation, Surgical):ti,ab,kw 307

#6 (Replantations, Surgical):ti,ab,kw OR (Surgical Replantations):ti,ab,kw OR (Surgical Replantation):ti,ab,kw 35

#7 #4or#5or#6 307

#8 MeSH descriptor: [Necrosis] explode all trees 19107

#9 (Necrosis):ti,ab,kw 21555

#10 #8or#9 39226

#11 #3and#7and#10 5

**Web of science**

| TS=(Fingers) OR TS=(Finger) OR TS=(Thumb) OR TS=(Digital) | 1194282 |
| --- | --- |
| TS=(Replantation) OR TS=(Replantations) OR TS=(Reimplantation) OR TS=(Reimplantations) OR TS=(Replantation, Surgical) OR TS=(Replantations, Surgical) OR TS=(Surgical Replantations) OR TS=(Surgical Replantation) | 13878 |
| TS=(Necrosis) | 490286 |
| #1 AND #2 AND #3 | 86 |


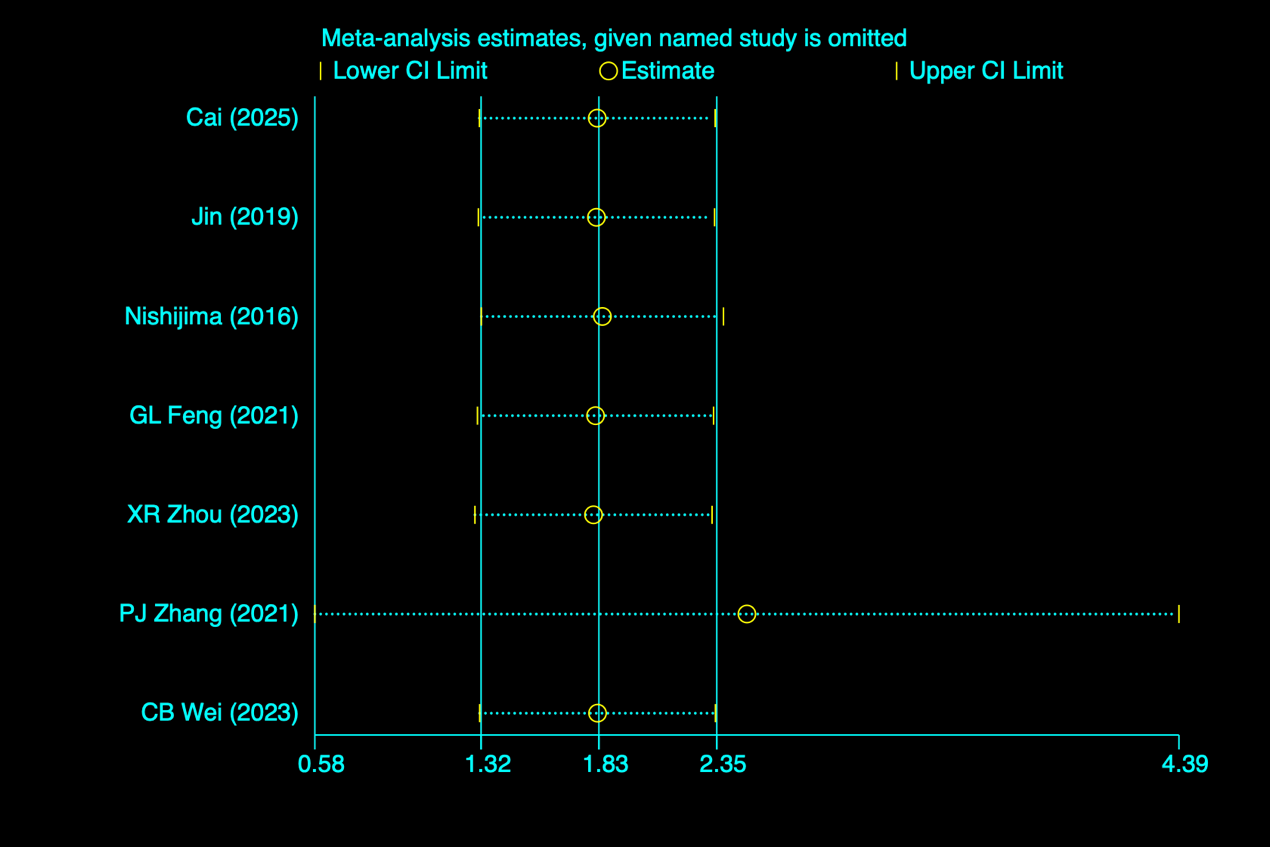


Figure S1 smoking Sensitivity Analysis


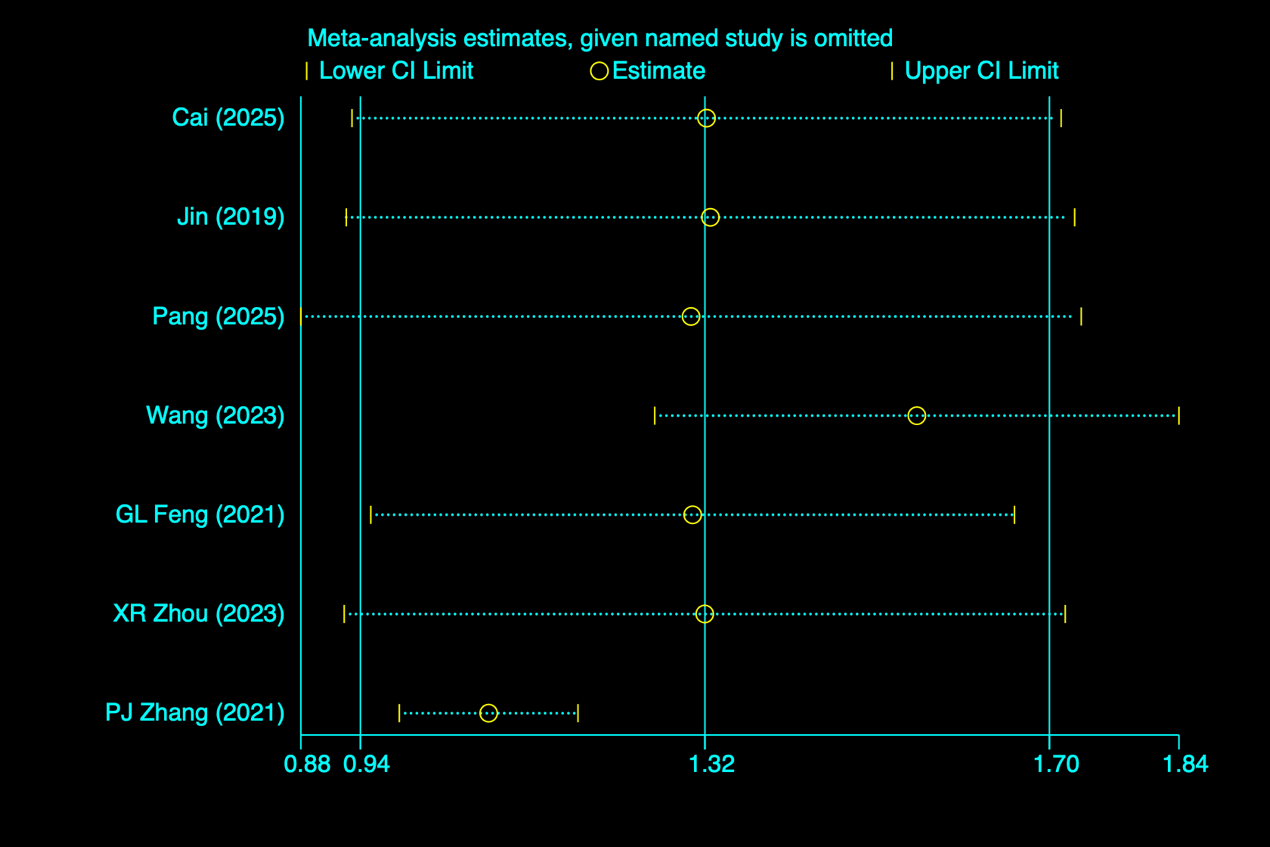


Figure S2 preoperative ischemic time ≥ 8h Sensitivity Analysis


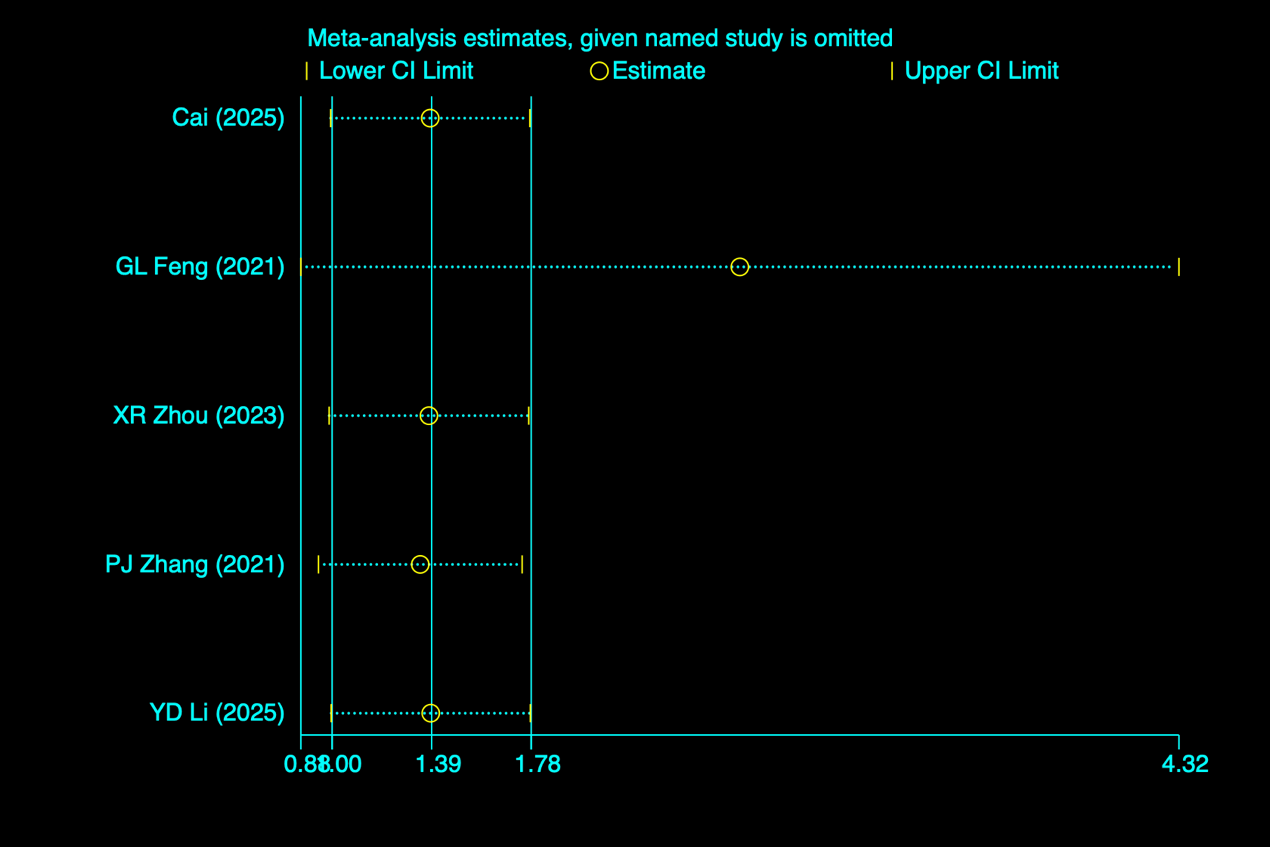


Figure S3 crush injury Sensitivity Analysis


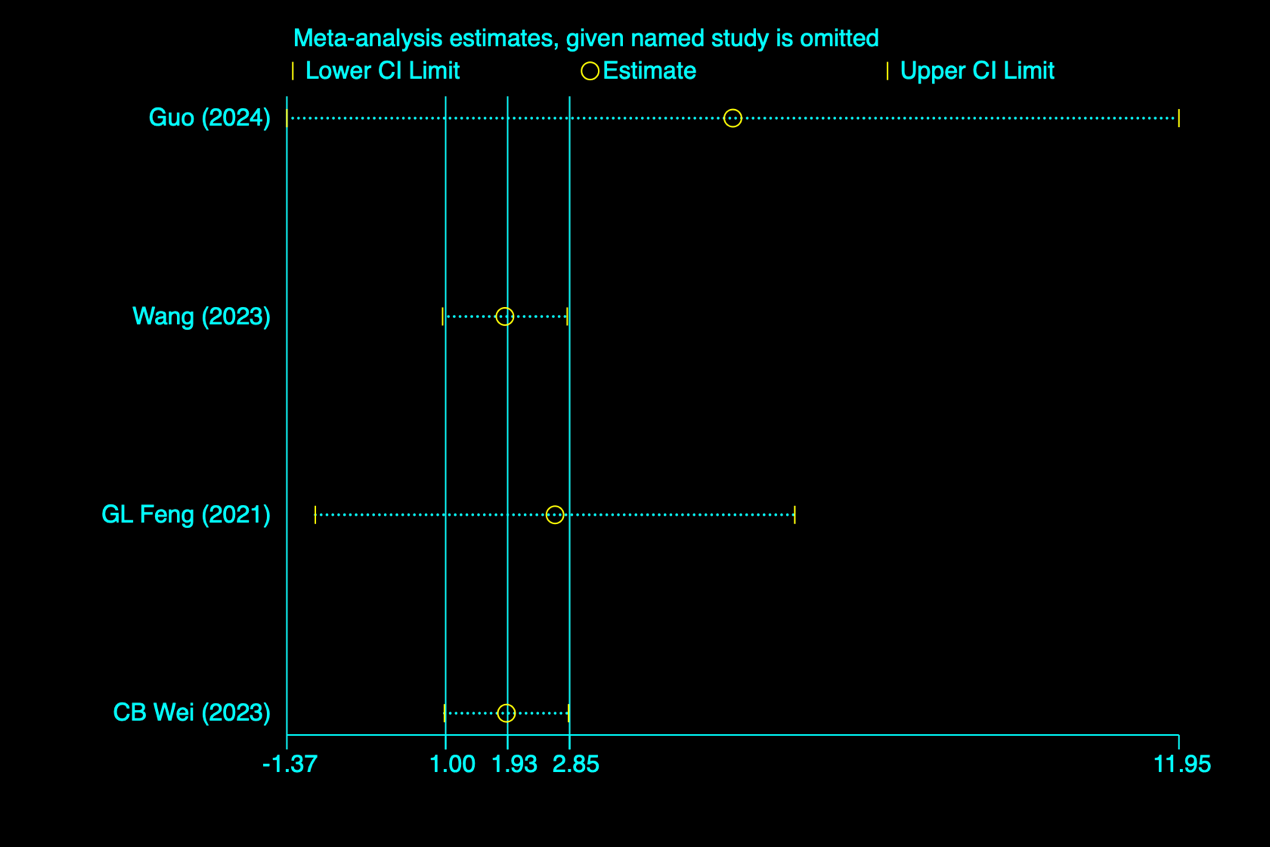


Figure S4 thrombosis Sensitivity Analysis


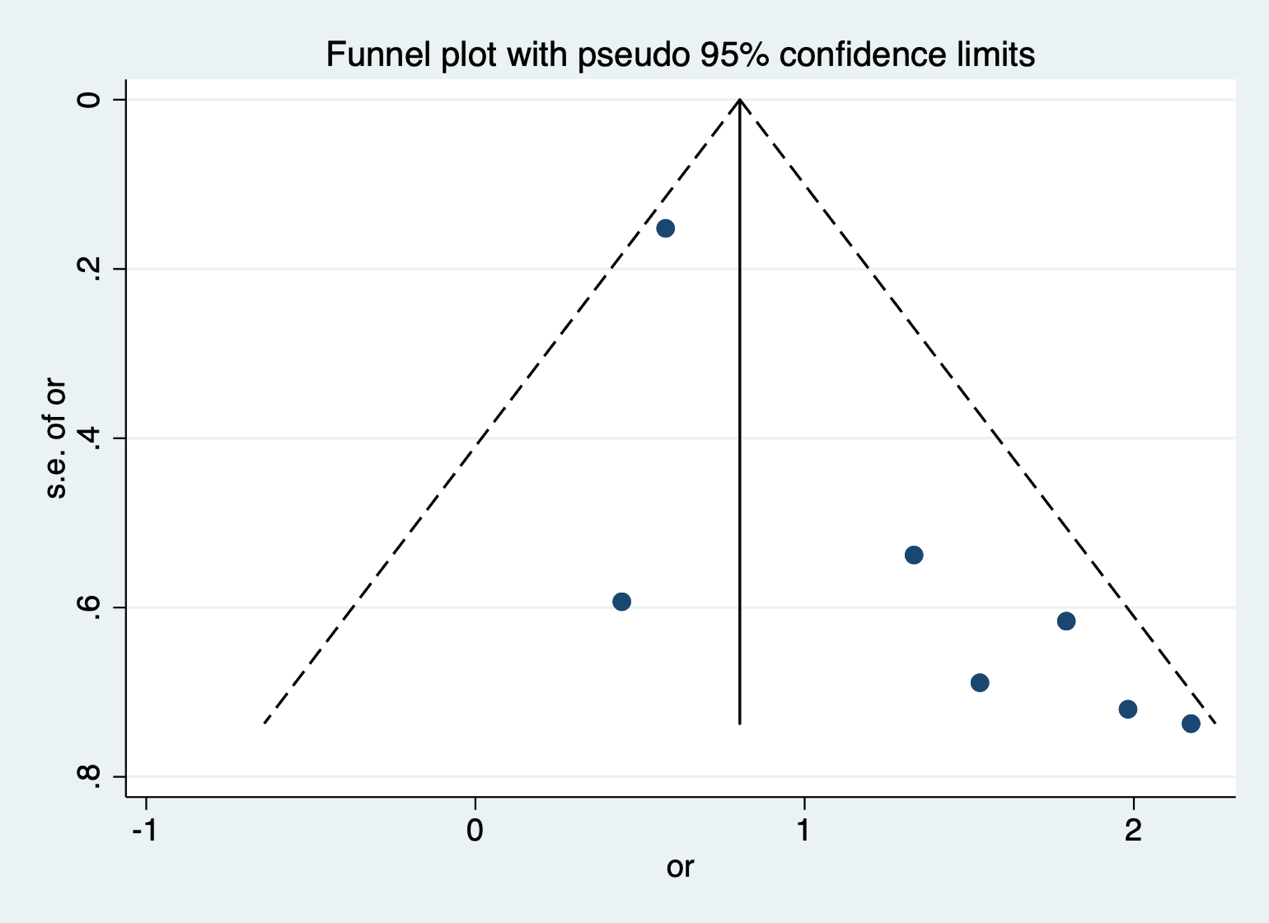


Figure S5 Funnel plot of meta-analysis on smoking


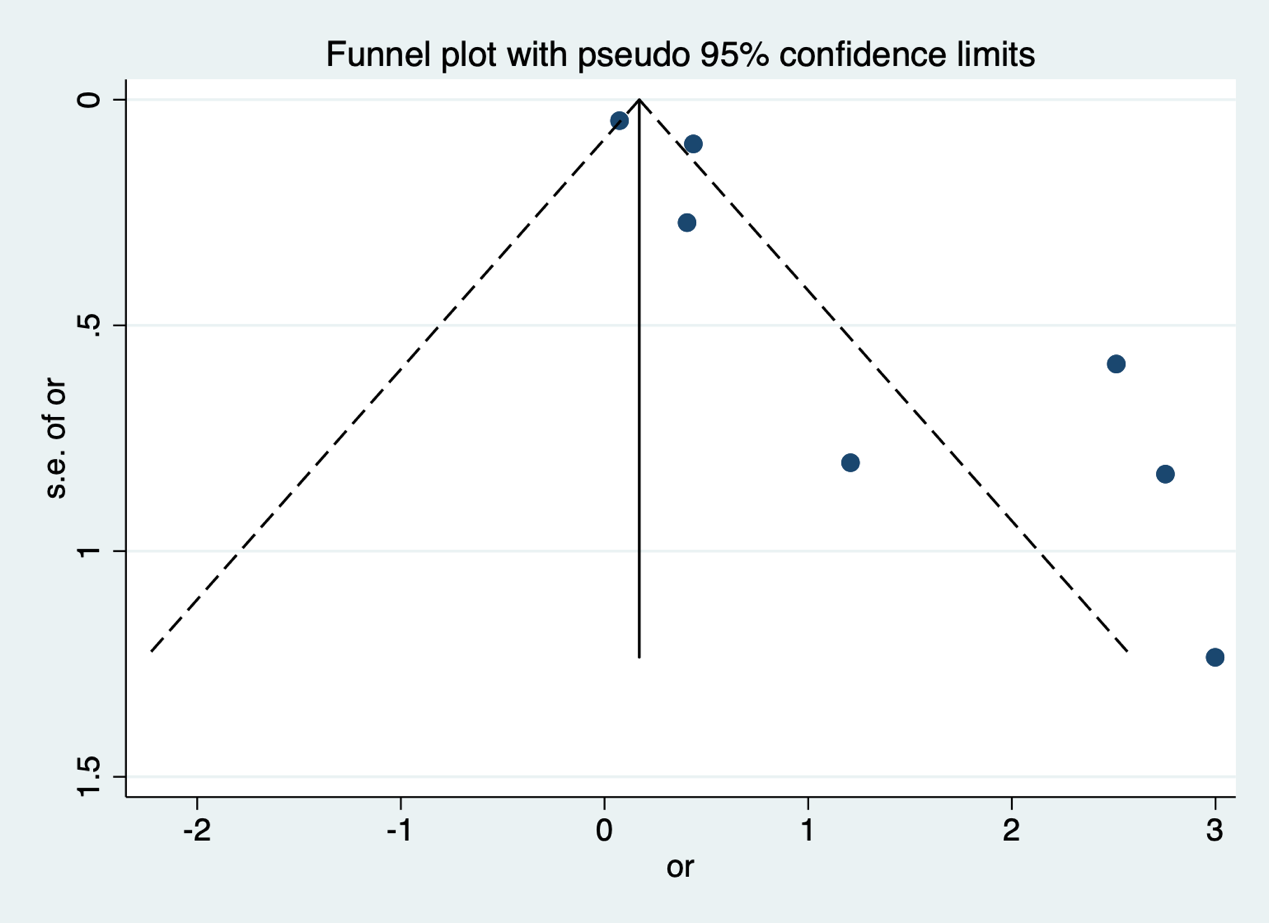


Figure S6 Funnel plot of meta-analysis on preoperative ischemic time ≥ 8h


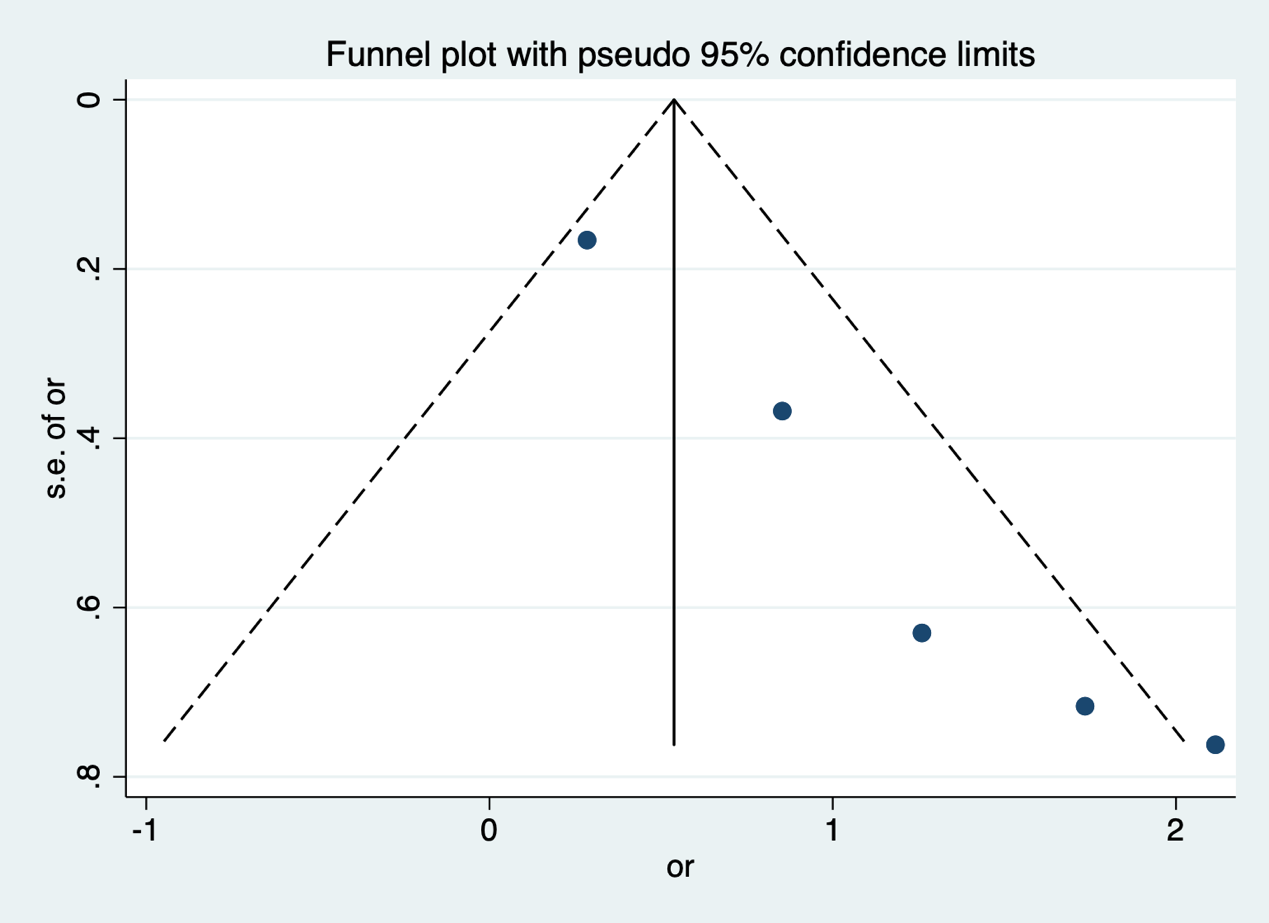


Figure S7 Funnel plot of meta-analysis on crush injury


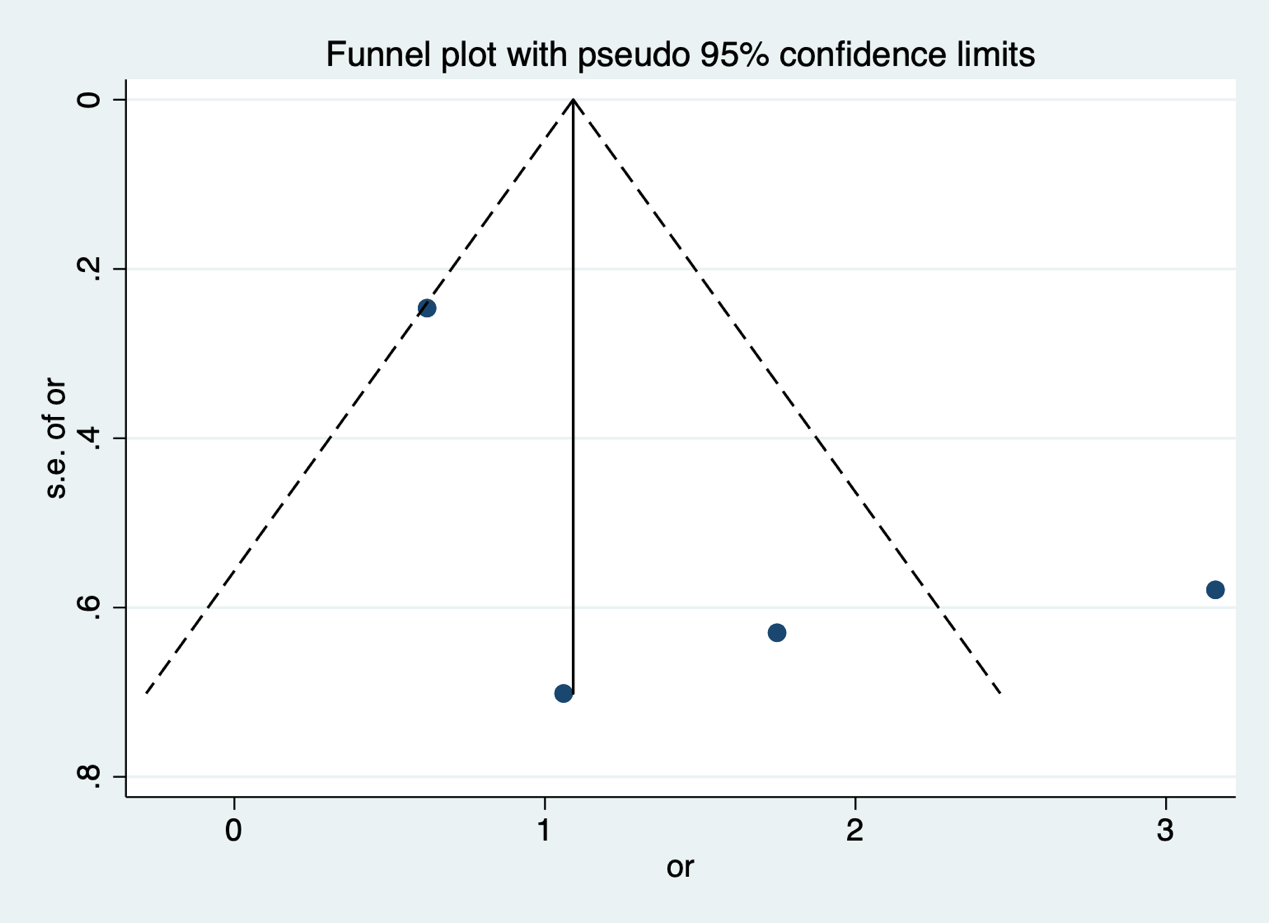


Figure S8 Funnel plot of meta-analysis on thrombosis


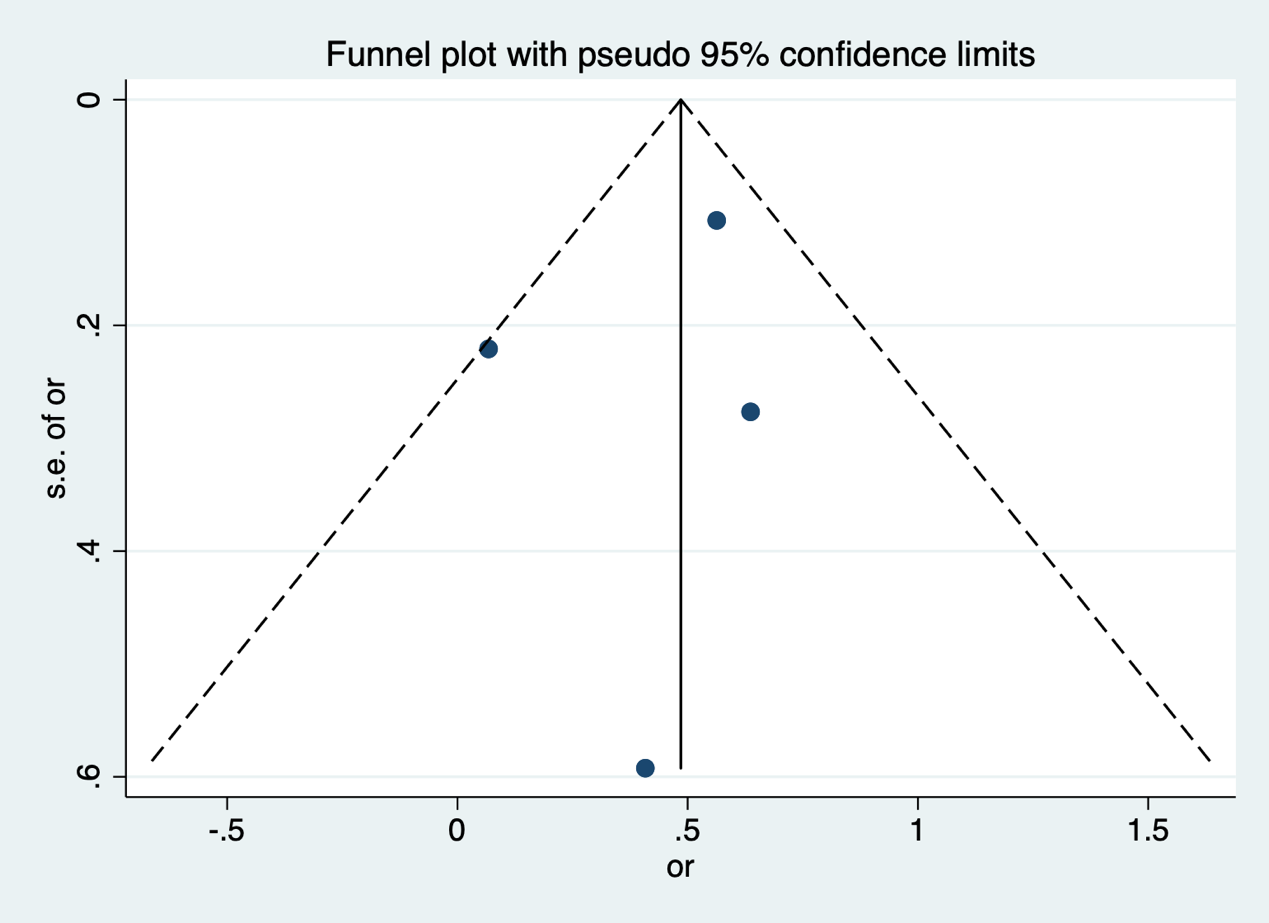


Figure S9 Funnel plot of meta-analysis on vein graft


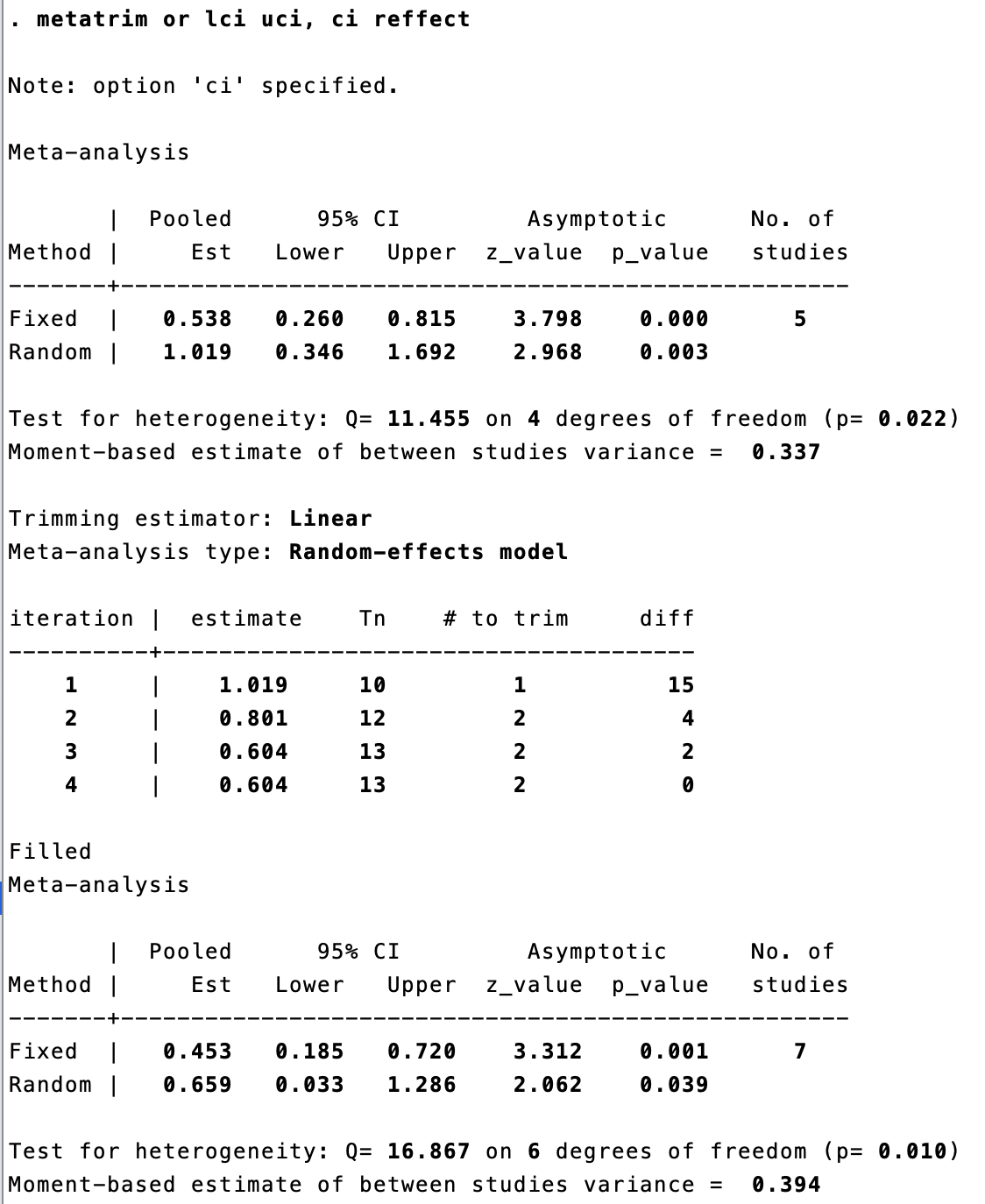


Figure S10 trim-and-fill results of the crush injury meta-analysis


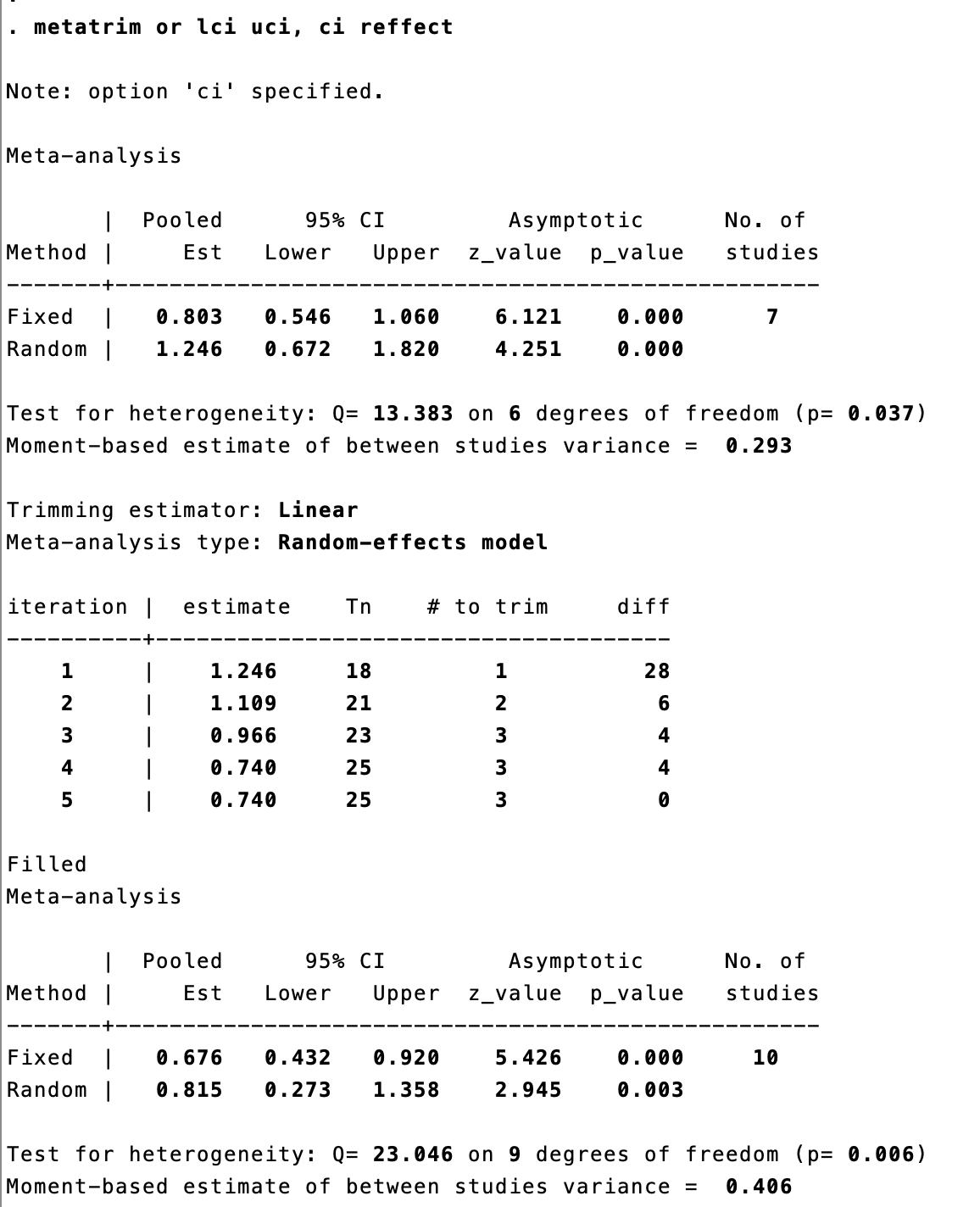


Figure S11 trim-and-fill results of the smoking meta-analysis


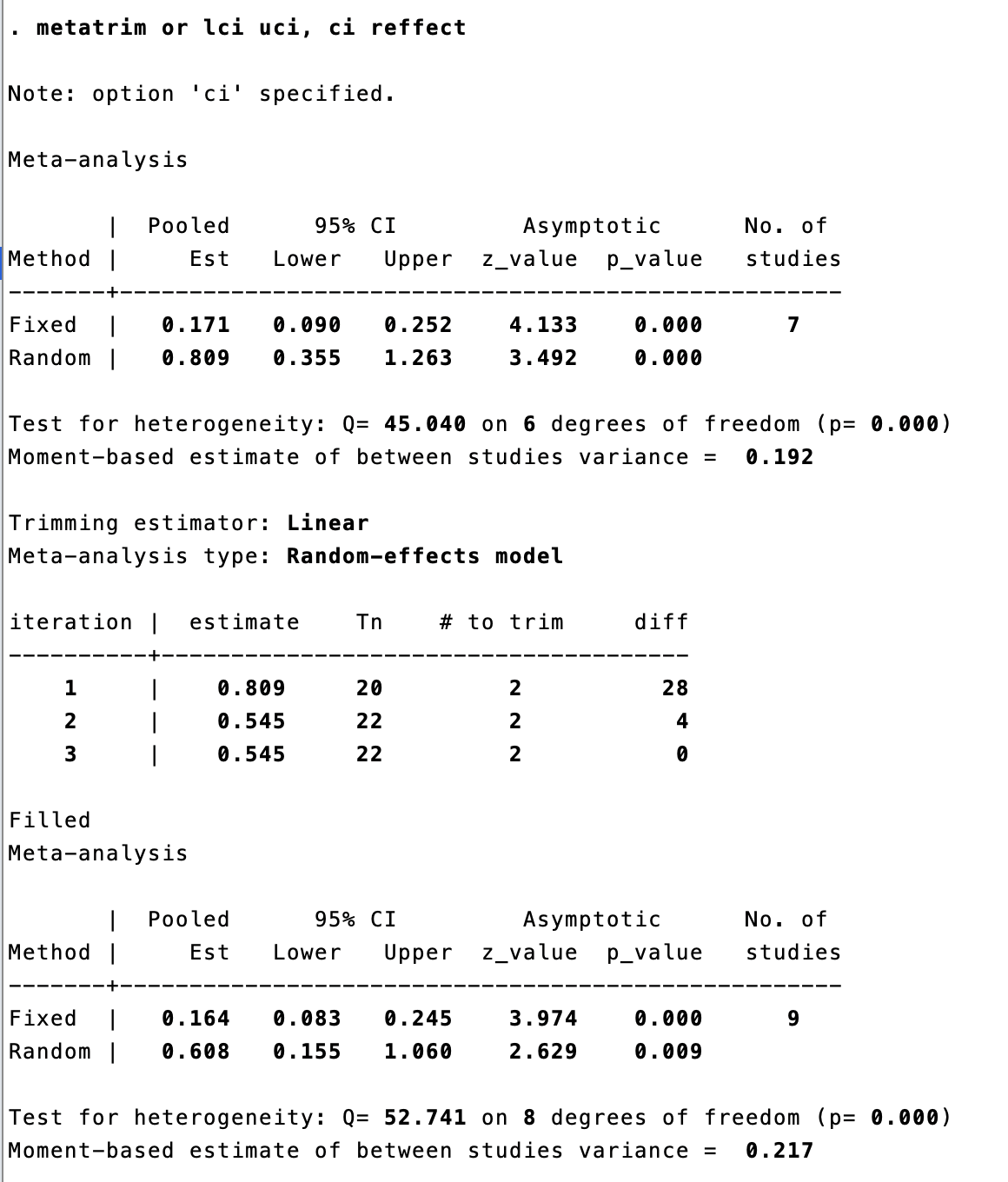


Figure S12 trim-and-fill results of the preoperative ischemic time ≥ 8h meta-analysis
